# The Effects of Mepolizumab on CRSwNP: Real-Life Evidence

**DOI:** 10.3390/jpm14111112

**Published:** 2024-11-20

**Authors:** Elena Cantone, Bernardino Cassiano, Paolo Pezzella, Mario Brandon Russo, Aikaterini Detoraki

**Affiliations:** 1 Department of Neuroscience, Reproductive Sciences and Dentistry, University of Naples “Federico II”, 80138 Naples, Italy; ppezzella@gmail.com; 2 UOSD di Otorinolaringoiatria, ASL Napoli 3, Ospedale di Nola, Via delle Repubbliche 7, 80035 Nola, Italy; 3 Department of Engineering, University of Campania “Luigi Vanvitelli”, 81031 Aversa, Italy; 4 Division of Internal Medicine and Clinical Immunology, Department of Translational Medical Sciences, University of Naples “Federico II”, 80138 Naples, Italy

**Keywords:** CRSwNP, biologics, anti-IL5, QoL, asthma, olfaction, VAS, nasal polyps

## Abstract

**Background**: This study aims to evaluate the efficacy and safety of mepolizumab in the treatment of severe uncontrolled CRSwNP with or without comorbid asthma in a real-life setting over the first six months of therapy. **Methods**: A total of 45 patients with nasal polyps with or without comorbid asthma were treated with mepolizumab (100 mg q4w) for 6 months. The following outcomes were assessed before therapy (V^0^), and after 6 months (V^1^): endoscopic nasal polyp score (NPS), nasal congestion score (NCS), sinonasal outcome test (SNOT-22), visual analog scale (VAS), nasal flow rate (PNIF), olfactory test (SS-I), and asthma control test (ACT). Blood eosinophil count, oral steroid intake, and rescue surgery were also measured. **Results**: We found a statistically significant improvement in NPS, NCS, SNOT-22, overall VAS, PNIF, SS-I, and ACT. In addition, we observed a decrease in blood eosinophils count. Mepolizumab was well tolerated, and no patients interrupted the treatment during the follow up. **Conclusions**: Our real-life study confirmed the efficacy and tolerance of mepolizumab prescribed for CRSwNP with or without asthma. The safety profile of mepolizumab was consistent with previous reports.

## 1. Introduction

Chronic rhinosinusitis with nasal polyps (CRSwNPs) is a chronic sinonasal inflammatory disease that strongly affects patients’ quality of life (QoL) and places a significant economic burden on national healthcare systems [1].

CRSwNP is a heterogeneous disease, whose pathogenesis is mainly mediated by the type 2 (T2) inflammatory pathway driven by allergic or non-allergic mechanisms in Western countries [2]. Conversely, emerging evidence focused on mixed type 1 and 3 (Th1 and Th17) immune responses and tissue neutrophilia involved in CRSwNP patients in some Asian countries [3].

T2 inflammatory responses are triggered, maintained, and amplified by synergistic interactions between the innate and adaptive immune systems [2]. The T2 pathway is mediated by innate lymphoid cells of group 2 (ILC2) and T helper 2 (Th2) lymphocytes, which produce and secrete T2 cytokines as interleukins 4 (IL-4), 13 (IL-13), and 5 (IL-5) [2].

IL-5 guides the maturation, proliferation, differentiation, and activation of eosinophils and inhibits the apoptotic death [2].

Other cells involved in the T2 inflammatory cascade are tissue-resident memory T cells (Trm), T follicular helper 2 (Tfh2) and 13 (Tfh13) cells, mast cells, and basophils [2]. In this scenario, the dysregulation of airway epithelium, promoted by pathogenic agents (aeroallergens, pollution, smoking, and viruses and bacteria), plays a crucial role [2]. These pathogens damage sinonasal epithelial cells and stimulate alarmins [2], like thymic stromal lymphopoietin (TSLP), interleukin-25 (IL-25), and interleukin-33 (IL-33), acting as triggers of immune mechanisms of type 2 inflammation [2].

T2 inflammation has been described as a common pathophysiological mechanism of CRSwNP and several comorbidities, such as asthma, allergic rhinitis, atopic dermatitis, etc., which often coexist in the same patient [4,5,6].

From a therapeutic point of view, the standard of care for CRSwNP includes intranasal steroids (INSs), short courses of oral corticosteroids (OCSs), and endoscopic nasal surgery (ESS).

INS sprays are one of the most widely used treatments for long-term medical therapy, while OCSs are recommended for exacerbations and relapses [7]. OCSs are characterized by many systemic adverse effects, so high doses and their prolonged use are not recommended in CRSwNP [7]. In addition, since the clinical practice is characterized by heterogeneity in terms of type, dosage, and duration of OCSs, there is no universally accepted protocol for their prescription [7].

ESS is the gold standard treatment for CRSwNP refractory to adequate medical therapy (AMT). It is associated with rapid improvement of symptoms and seems to optimize the efficacy of INSs. However, relapse occurs at a variable and non-predictive rate, and patients often require multiple surgical interventions [8].

Although the standard of care is widely used for CRSwNP, many patients do not respond to AMT and surgery, and relapse of nasal polyps is common [1]. Therefore, new treatment options are needed.

Recently, several biologics have been approved for the treatment of CRSwNP and T2 comorbidities [9,10]. Biologics directed against interleukin IL-4, IL-13, IL-5, and IgE are of high clinical interest, particularly in patients with severe uncontrolled CRSwNP [1,2,10].

Mepolizumab is a humanized IgG1/kappa class monoclonal antibody (mAb) selectively targeting human interleukin-5 (IL-5), a cytokine implicated in the recruitment, differentiation, survival, and degranulation of eosinophils, which play a crucial role in airways inflammation [8]. Subcutaneous injection of mepolizumab every 4 weeks has been approved in Italy for severe eosinophilic asthma (SEA) in 2015, and more recently in 2023 for CRSwNP [11,12].

The efficacy and safety of mepolizumab for the treatment of CRSwNP have been already demonstrated in a randomized clinical trial (RCT), post hoc analyses, and real-life studies [4,9,13,14].

This study aimed to evaluate the efficacy and safety of mepolizumab in the treatment of uncontrolled CRSwNP with or without comorbid asthma in a real-life setting over the first 6 months of treatment.

## 2. Materials and Methods

In this real-life study weincluded patients with CRSwNP referred to the Departments of Neuroscience, Reproductive Sciences and Dentistry, and Translational Medical Sciences of the University of Naples “Federico II”.

We enrolled consecutive patients treated between July 2023 and July 2024, collecting data at baseline and follow up.

According to both the Italian Agency of Drugs (AIFA) guidelines and the EPOS/EUFOREA update, patients eligible for this study were ≥18 years old with severe CRSwNP [nasal polyps score (NPS) ≥ 5 and/or sinonasal outcome test (SNOT-22) ≥ 50] bilateral T2 (confirmed by blood eosinophil counts > 150 cells/μL or tissue eosinopils ≥ 10/HPF or total IgE ≥ 100), inadequate symptom controls with INSs, failure (or intolerance) of previous medical treatments (at least two cycles of systemic corticosteroid over the last year), and/or previous ESSs [1,12,15].

The eventual coexistence of asthma was ascertained according to the 2022 Global Initiative for Asthma’s definition and ERS guidelines [16,17].

Exclusion criteria were pregnancy, treatment with another biologic drug in the current or previous 6 months, immunosuppressive treatment, radiotherapy or chemotherapy in the current or previous 12 months, and long-term steroid therapy for chronic autoimmune conditions.

This study was conducted following the Declaration of Helsinki and was approved by the Institutional Review Board of “Federico II” University Hospital (Prot.75/21, data of approval: 6 May 2021).

According to the AIFA guidelines, mepolizumab was administered through 100 mg subcutaneous injection every four weeks as add-on therapy to INSs [12].

The first administration was supervised by an ear, nose, and throat (ENT) physician, with subsequent self-administration by patients. The follow up was conducted at our hospital every 3 months. Treatment success was measured according to the criteria outlined in the EPOS [1].

We reported data at baseline (V^0^) and at 6 months (V^1^) follow up. Patients underwent the nasal endoscopy (the polyps were scored using the NPS), the nasal congestion score (NCS), the self-assessment of the disease-related QoL by the SNOT-22 questionnaire, and the visual analog scale (VAS) assessment for nasal obstruction (VASo), smell function (VASs), and rhinorrhea (VASr) [1,9,18,19,20].

We also reported the overall VAS symptom score combining scores for nasal obstruction, rhinorrhea, facial pain, and loss of smell [5].

The disease was divided into mild, moderate, and severe based on total severity VAS scores (mild, VAS = 0–3; moderate, VAS = 4−7; and severe, VAS = 8−10). A VAS > 5 affected patients’ QoL [1].

The nasal endoscopy was performed with a 2.7 mm 30-degree rigid endoscope (Storz, Tuttlingen, Germany). Trained ENT physicians assessed the NPS. The volume of polyps was measured under endoscopic view at each side of the nasal cavity using a 0–4 score; a higher score represented a larger volume of polyps [5].

For NCS evaluation, the patients were asked to assess their degree of nasal congestion on a 0- to 3-point score; a higher score represented a higher nasal congestion.

The SNOT-22 is a validated patient self-report questionnaire encompassing all major symptoms of CRS [19].

Patients rate the severity of 22 symptoms on a six-point Likert scale. The total score ranges from 0 to 110, with higher scores indicating a lower CRS-related QoL. The 22 questions are divided into four domains: nasal symptoms, ear and facial symptoms, sleep function, and psychological problems [19].

Asthma control was assessed through the asthma control test (ACT) [1,4].

The ACT measures asthma symptom control using 5 items on a 5-point score from 1 to 5, and the main outcome was the total score [21].

The nasal flow rate was measured by the Inspiratory Flow Meter In-Check Nasal (PNIF) (Clement Clarke International Ltd., Essex, UK) to objectify nasal obstruction. Three maximal inspirations were obtained, and the highest of the three measurements was considered for the assessment [22].

The olfactory function was evaluated by the standardized Sniffin’ sticks odor identification test (SS-I) 16 odor set test (Burghart Company, Wedel, Germany).

Odor identification was assessed for 16 odors. A multiple choice identification task of individual odors was performed from lists of four descriptors each. The subjects’ scores ranged from 0 to 16 [23].

Serum eosinophil count and total IgE, the number of past surgeries, comorbidities [asthma, nonsteroidal anti-inflammatory drug-exacerbated respiratory disease (NSAID-ERD)], and adherence to add-on therapy were also evaluated. In addition, we assessed rescue oral corticosteroids (OCSs) and surgery during the follow up.

### Statistical Analysis

Data were analyzed using Matlab R2021b 2024a and Microsoft Excel v.16/91. We described clinical and demographic characteristics with the appropriate descriptive statistics indexes. Descriptive statistics determined means and standard deviations (SDs) for symptom quantifications, comparing them to baseline significance using Student’s t-test for normally distributed data and the Mann–Whitney-U test for asymmetric distributions, with a significance level set at *p* < 0.05.

## 3. Results

We enrolled 45 patients (age: 59.7 ± 15.2 SD), mainly males (32 males, 71%; 13 females, 29%). Asthma was present in twenty-one (47%) and NSAID-ERD in eight (18%) patients. Thirty-three (73%) patients had received more than two cycles of OCSs throughout the last year, and forty-two (93%) had undergone at least one previous ESS, but no patient reported more than two surgical interventions. Baseline characteristics are reported in Table 1.

**Figure 1:**
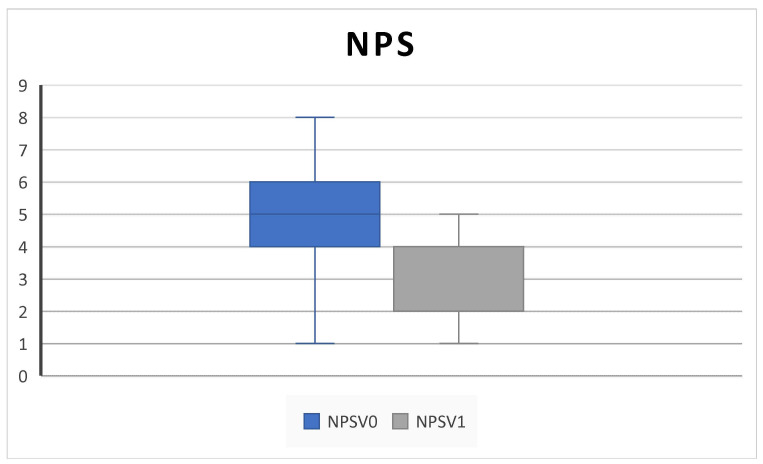
NPS (nasal polyp score) decreases after 6 months of therapy (V^0^: baseline; V^1^; 6 months) (5.2 ± 3.2 SD to 2.5 ± 1.4 SD; *p* = 0.004).

**Figure 2:**
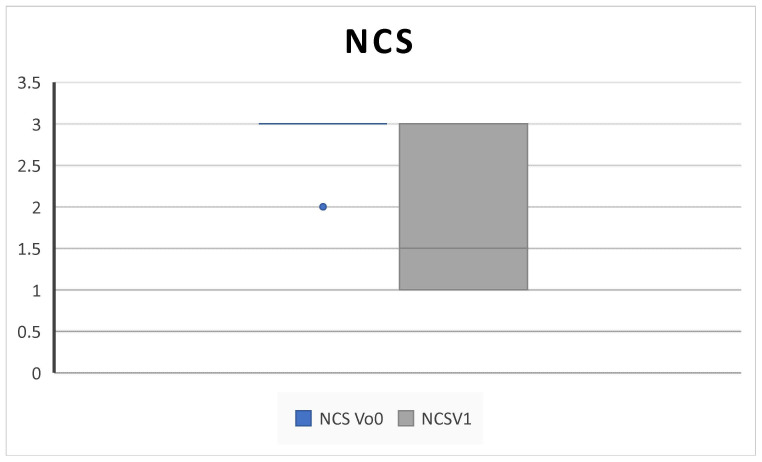
NCS (nasal congestion score) decreases after 6 months of therapy (V^0^: baseline; V^1^; 6 months) (2.8 ± 0.3 SD to 1.8 ± 0.9 SD; *p* = 0.02).

**Figure 3:**
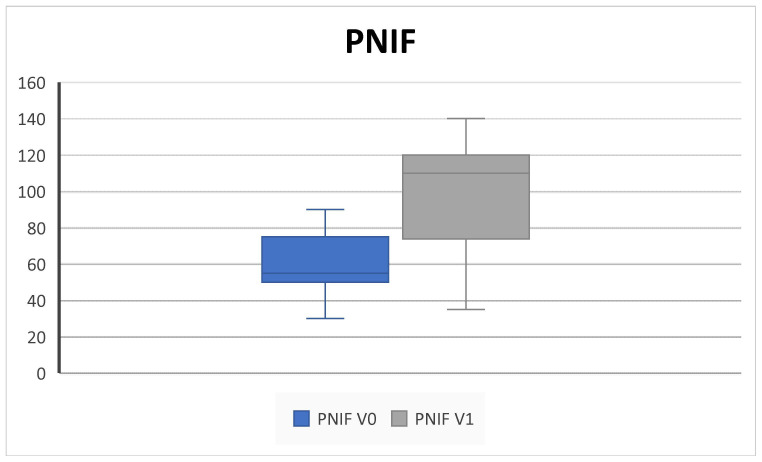
PNIF (pick nasal inspiratory flow) decreases after 6 months of therapy (V^0^: baseline; V^1^; 6 months) (58.7 ± 18.8 SD to 100 ± 33.9; *p* = 0.009).

All patients completed at least 6 months of follow up. We collected outcome measures for NPS in 45/45 patients at baseline and 42/45 at 6-month follow up, NCS in 38/45 patients at baseline and 40/45 at 6-month follow up, PNIF in 39/45 patients at baseline and 35/45 at 6-month follow up, SNOT-22 and VAS in 45/45 patients at baseline and 43/45 at 6-month follow up, EOS in 43/45 patients at baseline and 40/45 at 6-month follow up, and SS-I in 22/45 patients at baseline and 20/45 at 6-month follow up due to the unavailability of the test. We observed a significant improvement in NPS (5.2 ± 3.2 SD to 2.5 ± 1.4 SD; *p* = 0.004), NCS (2.8 ± 0.3 SD to 1.8 ± 0.9; *p* = 0.02), and PNIF score (58.7 ± 18.8 SD to 100 ± 33.9; *p* = 0.009) (Figure 1, Figure 2 and Figure 3).

**Figure 4:**
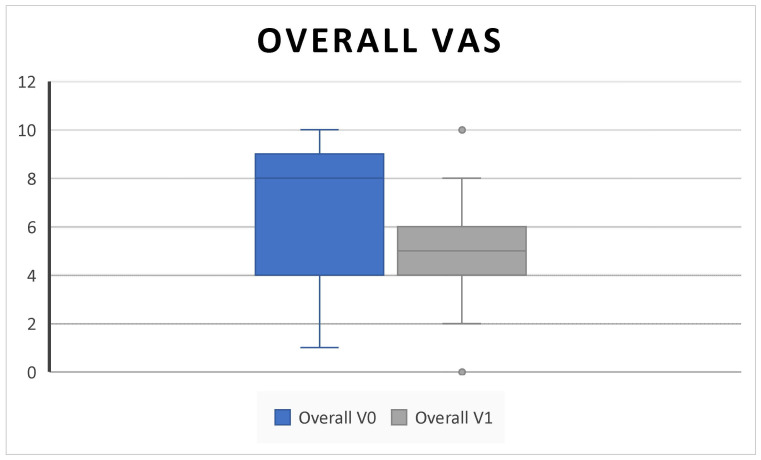
The overall VAS (the overall VAS symptoms score combines scores for nasal obstruction, rhinorrhea, facial pain, and loss of smell) decreases after 6 months of therapy (V^0^: baseline; V^1^; 6 months) (6.53 ± 3.1 SD to 4.8 ± 2 SD; *p* = 0.02).

In addition, we found a significant decrease in the overall VAS score (6.53 ± 3.1 SD to 4.8 ± 2 SD; *p* = 0.02) (Figure 4).

We did not find statistically significant improvements in subjective VAS evaluation for each symptom: rhinorrhea (*p* = 0.7), nasal obstruction (*p* = 0.1), and smell (*p* = 0.1).

**Figure 5:**
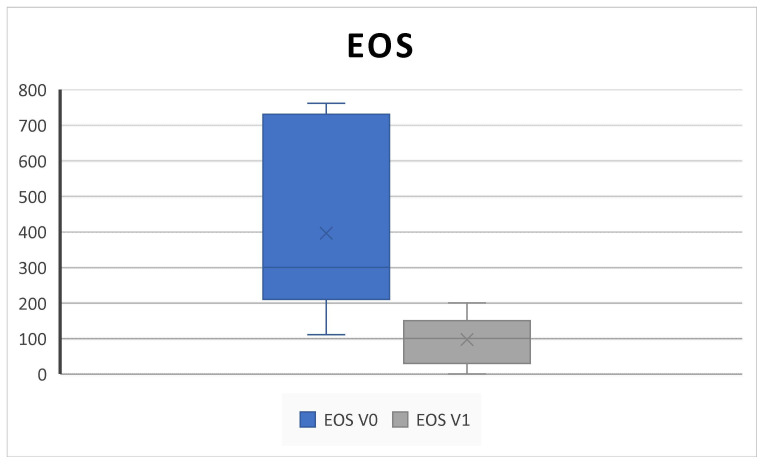
The blood eosinophil (EOS) count decreases after 6 months of therapy (V^0^: baseline; V^1^; 6 months) (421.0 ± 302.7 SD to 75.0 ± 71.4 SD; *p* = 0.001).

Blood eosinophil count significantly decreases from 421.0± 302.7 SD to 75.0± 71.4 SD (*p* = 0.001) (Figure 5).

**Figure 6:**
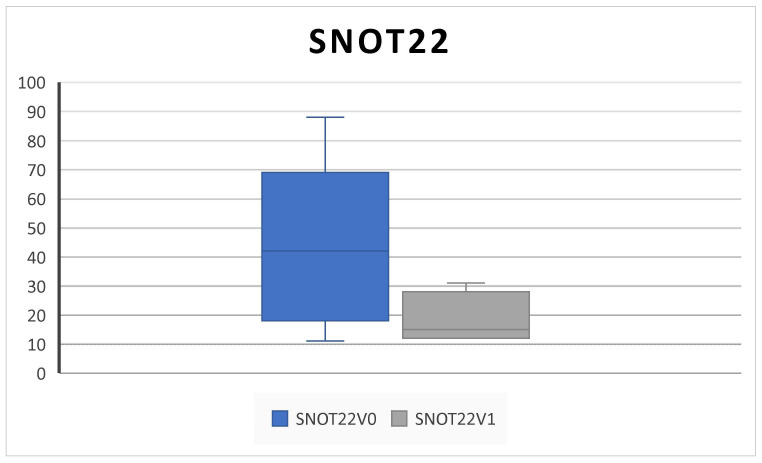
QoL improvement. The SNOT-22 (sinonasal outcome) decreases after 6 months of therapy (V^0^: baseline; V^1^; 6 months) (61.3 ± 24.1 SD to 19.5 ± 8.4 SD; *p* = 0.001).

Patients’ QoL assessed by the SNOT-22 score significantly improved from 61.3 ± 24.1 SD at V^0^ to 19.5 ± 8.4 SD at V^1^ (*p* = 0.001) (Figure 6).

**Figure 7:**
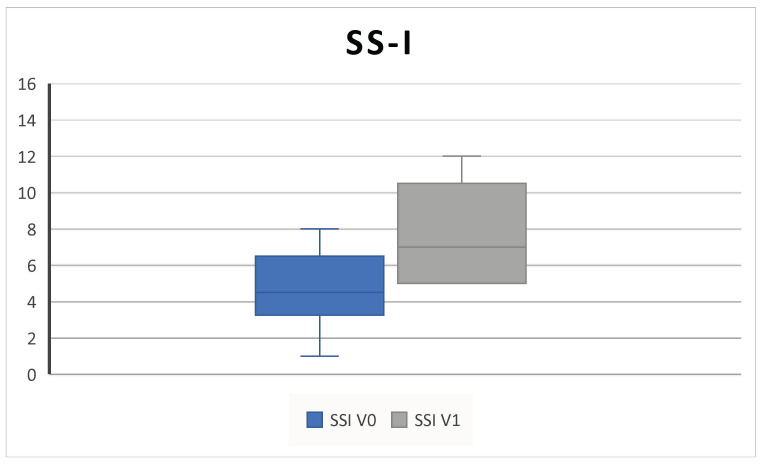
The SS-I (sniffin’ sticks identification test) increases after 6 months of therapy (V^0^: baseline; V^1^; 6 months) (4.1 ± 2.3 SD to 7.5 ± 2.8 SD; *p* = 0.02).

In the group of patients that performed SS-I, whose characteristics (Table 2) did not differ from the rest of the study population (*p* > 0.05 for all parameters), we found an improvement in the SS-I score from 4.1 ± 2.3 SD at V^0^ to 7.5 ± 2.8 SD at V^1^ (*p* = 0.02) (Figure 7).

In the subgroup of patients with asthma, we observed a statistically significant (*p* = 0.009) improvement of ACT from 15.4 ± 5.5 SD to 24.0 ± 1.2 SD.

In addition, we did not find significant differences between asthmatic and non-asthmatic patients after treatment. In particular, we evaluated the reduction in nasal polyps (NPS: 2.7 ± 1 SD vs. 2.4 ± 1 SD, *p* = 0.4), the nasal flow rate (PNIF: 98.8 ± 31 SD vs. 101.7 ± 24 SD, *p* = 0.7), and the QoL (SNOT-22: 19.2 ± 6 SD vs. 21.7 ± 7 SD, *p* = 0.5).

Adherence to INS add-on therapy was 83% (35/45). No patients interrupted mepolizumab and no patients required sinonasal surgery during the follow up. Only one patient with SEA required a cycle of OCSs.

Mepolizumab was well tolerated, and five (11.1%) patients reported pain, redness, or edema in the injection site within 24 h after the administration of biologic. One patient reported a headache that resolved spontaneously 5 days after the first administration (Table 3).

We did not find a correlation between the SNOT-22, NPS, NCS, PNIF, SSI, and level of blood EOS (*p* > 0.05) at baseline.

## 4. Discussion

A total of 80–90% of CRSwNP patients are characterized by high eosinophils count. Eosinophils mediate tissue damage and polyp growth due to the release of cytokines. Eosinophilic infiltration and activation are potentiated by IL-5, which is a potent indicator of eosinophilic chemotaxis, activation, and survival [4].

Mepolizumab is a humanized mAb that binds with high affinity to and inactivates IL-5, which promotes eosinophils recruitment. The phase III RCT study SYNAPSE demonstrated efficacy, good tolerability, and safety profile of 100 mg mepolizumab administered subcutaneously every 4 weeks as an add-on treatment to INSs for CRSwNP, and the AIFA approved mepolizumab for the treatment of CRSwNP in Italy in 2023 [8,11,19].

Although RCTs are crucial in developing a new drug, real-life studies are mandatory to evaluate the efficacy in clinical practice, considering the heterogeneity of the general population and the clinical characteristics that may influence outcomes.

So far, a limited number of studies have evaluated the efficacy of mepolizumab prescribed for CRSwNP [4,5,9,24].

In the study by Detoraki et al., the authors observed that mepolizumab improved sinonasal and asthmatic symptoms and reduced polyp growth in 44 patients with SEA and CRSwNP treated for 12 months, in which mepolizumab was prescribed for asthma. No VAS or olfactory function tests were performed [4].

In the study by Domínguez-Sosa et al., the authors demonstrated that mepolizumab prescribed for asthma improved the SNOT-22, NPS, and the overall VAS in 55 patients with both asthma and CRSwNP treated for 6 months. Moreover, they observed a significant reduction in blood eosinophil count. They did not evaluate olfactory function using a test [5].

Two recent Italian studies found an improvement in the clinical features of CRSwNP after 12 months of treatment [11,24].

In the first study, the authors demonstrated the efficacy of mepolizumab in promoting the reduction in nasal polyps, the decrease in blood eosinophils, and the improvement of disease-related symptoms and QoL in a small sample of 22 patients. They evaluated the VAS for smell but did not perform an olfactory test [11].

In the second study, the authors showed an improvement in the SNOT-22, NPS, and SSI in 30 patients treated with mepolizumab for 12 months. They also observed a reduction in blood eosinophil count. They did not perform a specific VAS for each symptom [24].

To our knowledge, our study represents the largest Italian cohort of patients (45) undergoing mepolizumab prescribed for CRswNP according to the AIFA indication.

CRSwNP is associated with a range of symptoms (nasal congestion, rhinorrhea, olfactory dysfunction) that have a significant impact on the QoL, including physical and mental health, work capacity, social and emotional functioning, and sleep disturbance, with substantial direct and indirect costs to the healthcare system [1,4]. Therefore, the management of a chronic inflammatory disease, like CRSwNP, is of fundamental importance not only from a clinical health point of view but also from a social and work one [1].

Consistent with the SYNAPSE and MERIT RCTs and with other clinical real-life studies, our results confirmed that subcutaneous administration of 100 mg mepolizumab every 4 weeks as add-on therapy to INSs is effective in improving QoL and clinical features in CRSwNP patients over 6 months of treatment [4,5,8,10,25,26]. Indeed, we found a statistically significant improvement in the SNOT-22, overall VAS score, NPS, NCS, and PNIF; in addition, we found a decrease in the level of EOS count after treatment (Figure 1, Figure 2, Figure 3, Figure 4, Figure 5, Figure 6 and Figure 7).

Interestingly, according to the literature, we found that improvements in PNIF exceeded the minimum clinically important difference (MCID) of 20 L/min, and the SNOT-22 exceeded the MCID of 8.9 units [25,27].

While we found an improvement in overall VAS, as reported by other researchers, we did not observe an improvement in VAS scores for each symptom, probably due to the small sample size and the short follow up [11,24].

Notwithstanding, it can be assumed that the patients showed a general improvement in clinical condition and could not attribute this improvement to one symptom rather than another. Another speculation is that the improvement of each symptom was gradual and not immediately and subjectively perceived.

A longer follow up would certainly have been necessary to verify these data [5].

Furthermore, we observed a statistically significant decrease in the number of eosinophils in the blood [7], thus also confirming the role that anti-IL5 plays in the control of inflammation. However, we found no correlation between the SNOT-22, NPS, NCS, PNIF, SS-I, and level of blood EOS at baseline. These data would demonstrate the role of blood EOS in defining T2 inflammation but not in the assessment of the severity of the CRSwNP.

Olfactory dysfunction is one of the most difficult-to-treat CRSwNP symptoms. It substantially impacts QoL and has significant effects on psychological health [12].

In this study, baseline SSI confirmed that patients had a substantially impaired pre-treatment sense of smell (Figure 7).

In the subgroup of patients that underwent the olfactory test, we found a significant improvement in SSI after 6 months of mepolizumab (Figure 7). Although they represent less than half of the study cohort, their baseline characteristics did not differ from the entire study population, so the tested subgroup was representative of the entire cohort.

These real-life data are particularly intriguing.

In the SYNAPSE-RCT, the authors found a modest olfactory improvement, probably, as they affirmed, due to the history of multiple surgeries [9,12]. Indeed, multiple endoscopic nasal surgeries could damage the olfactory neuroepithelium, reducing the sense of smell [9].

Although a high percentage (93%) of our patients underwent surgery, the number of interventions was lower than that of the patients in the SYNAPSE-RCT (Table 1).

This would explain the reason why the olfactory improvement of our cohort is particularly relevant and significant.

In the subgroup of patients with asthma (47%), we observed an improvement in the ACT score. In our cohort, less than half of the subjects had comorbid asthma, unlike other studies that reported higher rates and greater severity of asthma-related symptoms compared to our population. We found the efficacy of mepolizumab in the treatment of CRSwNP in both asthmatic and non-asthmatic patients [4,5,9,10,11,24].

These data are quite intuitive given that in some real-life studies reported in the literature, mepolizumab was mainly prescribed for SEA [4,5,9,10,11,24].

In a series of six retrospective cases of patients with uncontrolled SEA and concomitant CRSwNP, the authors find that mepolizumab improves the control of asthma but not nasal polyposis. However, this study has several limitations: mepolizumab was prescribed for SEA and there were only six patients who had non-homogeneous characteristics. Furthermore, the authors did not record CRS symptoms with the SNOT-22 or VAS and did not evaluate olfactory function with the olfactory test. Therefore, clinical outcomes were defined partly based on a predominantly anamnestic evaluation [28].

We also observed high adherence to the therapy, and no patients interrupted mepolizumab. These data confirmed the good tolerability of mepolizumab in the treatment of CRSwNP.

Five patients reported pain, redness, or edema in the injection site within 24 h after the administration of mepolizumab. One patient reported a headache that resolved spontaneously 5 days after the first administration. These data confirmed the safety profile of mepolizumab (Table 3). However, the correlation between adverse events and the administration of mepolizumab, especially for headaches, remains to be demonstrated.

This study’s limitations include the small sample size and the short duration of follow up. In addition, data on SSI were not available in all patients. Therefore, future studies are needed to confirm our results.

## 5. Conclusions

So far, few real-world studies have been published on the effect of mepolizumab in patients with severe uncontrolled CRSwNP with or without asthma. This study confirms the currently available data providing evidence that mepolizumab is effective, well tolerated, and safe in the treatment of CRSwNP. This therapy had a positive impact on T2 comorbidities, the need for OCSs and surgery, and QoL.

The improvement in real-life outcomes is consistent with that of the main RCT and the other real-life studies [4,5,9,10,11,24]. Additionally, the results appear better than those of the RCTs, especially concerning the olfactory function [10].

We believe that our real-life findings could have significant implications for the management of patients with CRSwNP, regardless of comorbid asthma in the clinical practice.

## Figures and Tables

**Table 1:** Baseline characteristics.

N.	45
**AGE**	59.7 ± 15.2 SD
**SEX**	32 M (71%); 13 F (29%)
**EOS**	421.0 ± 302.7 SD
**ALLERGY**	18 (40%)
**TOTAL IGE**	236.6 ± 190.6 SD
**SNOT-22**	61.3 ± 24.1 SD
**VASO**	5.7 ± 2.8 SD
**VASR**	4.0 ± 3.3 SD
**VASS**	6.2 ± 3.9 SD
**OVERALL VAS**	6.53 ± 3.1 SD
**NPS**	5.2 ± 2.2 SD
**NCS**	2.8 ± 0.3 SD
**PNIF**	58.7 ± 18.8 SD
**SSI**	4.1 ± 2.3 SD
**ASTHMA**	21 (47%)
**NSAID-ERD**	8 (18%)
**ACT**	15.5 ± 5.5 SD
**AT LEAST 2 CYCLES OF OCS/YEAR**	33 (73%)
**AT LEAST 1 PREVIOUS SURGERY**	42 (93%) (Mean 1.3 ± 1.3 SD)

EOS (eosinophils), SNOT-22 (sinonasal outcome), PNIF (peak nasal inspiratory flow), SS-I (sniffin’ sticks identification test), VASo (visual analog scale for nasal obstruction), VASs (visual analog scale for smell function rhinorrhea, VASr (visual analog scale for rhinorrhea), NSAID-ERD (nonsteroidal anti-inflammatory drug-exacerbated respiratory disease), ACT (asthma control test).

**Table 2:** Baseline characteristics of the SSI group.

N.	22
**AGE**	58.4 ± 14.3 SD
**SEX**	14 M (64%); 8 F (36%)
**EOS**	557.1 ± 274.5 SD
**ALLERGY**	8 (36%)
**TOTAL IGE**	259.8 ± 258 SD
**SNOT-22**	77.7 ± 9.6 SD
**VASO**	4.5 ± 2.7 SD
**VASR**	4.7 ± 2.7 SD
**VASS**	6.4 ± 3.2 SD
**OVERALL VAS**	7.0 ± 1.8 SD
**NPS**	5.7 ± 1.5 SD
**NCS**	2.4 ± 0.7 SD
**PNIF**	64.5 ± 20.6 SD
**SSI**	4.1 ± 2.3 SD
**ASTHMA**	10 (45%)
**NSAID-ERD**	3 (14%)
**ACT**	16.5 ± 3.9 SD
**AT LEAST 2 CYCLES OF OCS/YEAR**	15 (68%)
**AT LEAST 1 PREVIOUS SURGERY**	20 (90%) (Mean 1.6 ± 0.8 SD)

EOS (eosinophils), SNOT-22 (sinonasal outcome), PNIF (peak nasal inspiratory flow), SS-I (sniffin’ sticks identification test), VASo (visual analog scale for nasal obstruction), VASs (visual analog scale for smell function rhinorrhea, VASr (visual analog scale for rhinorrhea), NSAID-ERD (nonsteroidal anti-inflammatory drug-exacerbated respiratory disease), ACT (asthma control test).

**Table 3:** Rescue therapy and adverse events.

Rescue Therapy	No. (%)
OCS	1 (2.2%)
Surgery	0
**Adverse event**	**No. (%)**
Injection site	5 (11.1%)
Headache	1 (2.2%)

One (2.2%) patient required a cycle of OCSs. Five (11.1%) patients reported pain, redness, or edema in the injection, and one (2.2%) patient reported a headache.

## Data Availability

The data presented in this study are available upon reasonable request from the corresponding author.
